# Dynamic Infrared Thermographic Evaluation of Facial Thermal Response During Face Mask Wearing

**DOI:** 10.3390/s26020460

**Published:** 2026-01-09

**Authors:** Radostina A. Angelova, Maria Dimova

**Affiliations:** 1 Department of Hydroaerodynamics and Hydraulic Machines, Technical University of Sofia, 1000 Sofia, Bulgaria; mdimova@tu-sofia.bg; 2 Miracle Centre of Competence Lab “Intelligent Mechatronic Solutions in Textiles and Clothing”, Technical University of Sofia, 1000 Sofia, Bulgaria; 3 Centre for Research and Design in Human Comfort, Energy and Environment (CERDECEN), Technical University of Sofia, 1000 Sofia, Bulgaria

**Keywords:** infrared thermography, face masks, facial temperature, thermal response, sensor-based evaluation, inner canthus, heat transfer

## Abstract

**Highlights:**

**What are the main findings?**

- Dynamic facial temperature changes during mask use were captured using a four-stage infrared thermographic protocol.
- The inner canthus exhibited high sensitivity to temporal facial temperature variations.

**What are the implications of the main findings?**

- Infrared thermography enables non-contact, protocol-based assessment of dynamic facial thermal behaviour.
- The inner canthus is a suitable reference region for thermographic facial measurements.

**Abstract:**

The study proposes a sensor-based experimental protocol for quantifying dynamic facial temperature changes during face mask use by means of infrared thermography (IRT). Eight face masks, including filtering respirators, surgical masks, and one textile mask, were evaluated on three participants under controlled indoor conditions. Thermographic data were acquired at four defined measurement stages: prior to mask application, immediately after donning, after 15 min of continuous wear, and immediately after removal. The measurements reveal a reproducible temporal temperature pattern across participants and mask types, consisting of an initial cooling phase, subsequent heat accumulation during wear, and a pronounced temperature increase following removal. Thermal variations were observed both in mask-covered and uncovered facial regions. The inner canthus exhibited high sensitivity to these changes, supporting its use as a stable reference area. The study demonstrates the suitability of IRT for protocol-driven, non-contact assessment of dynamic facial thermal response during mask use.

## 1. Introduction

Infrared thermography (IRT) is a non-contact, non-invasive technique, based on the physical principle that all bodies with a temperature above absolute zero emit electromagnetic energy in the infrared spectrum. The intensity of this radiation is directly related to the surface temperature and can be measured and visualised as a temperature field [1].

The total radiant emission of an ideal body (the so-called “black body”) is defined by the Stefan–Boltzmann law [2]:

(1) $$M={\sigma T}^{4}$$

where M is the radiant power (W/m^2^), T is the absolute temperature (K), and σ = 5.67 × 10^−8^ W/m^2^·K^4^ is the Stefan–Boltzmann constant.

Real objects emit less thermal energy than an ideal black body because they partially absorb or reflect the received energy, and in the case of semi-transparent materials, such as certain textile fabrics or glass, they transmit part of the radiation [3]. The reduction of thermal energy emitted by real bodies, compared to an ideal black body at the same temperature, is accounted for by the emissivity coefficient ε [1]. This coefficient indicates the fraction of energy that is actually emitted, and its value ranges between 0 and 1 (1 corresponds to a perfect black body) [1].

For human skin, ε is extremely high, between 0.96 and 0.99 [4], while for most textile materials (commonly used in face masks, such as polyester- or polypropylene-based fabrics) it is approximately 0.88 [5]. This means that skin emits almost 100% of its own heat, which is why infrared cameras (IC) can measure facial skin temperature with high accuracy, without the need for contact sensors or complex surface-property corrections. The emissivity of textiles (e.g., face masks) is also very high compared to many other engineering materials, due to their fibrous, light-diffusing and non-metallic structure [6]. Therefore, reliable infrared thermographic measurements of both face mask surfaces and facial skin are possible.

IRT is particularly suitable for studying thermophysiological comfort in the interaction between the human body and protective face masks [7,8]. IC can convert the thermal energy emitted by the human face into thermal images (thermograms), where each point of the face is visualised as a colour corresponding to a specific temperature [9]. In modern infrared imaging systems, the spatial resolution typically ranges from several hundred to thousands of pixels, while the thermal sensitivity can reach values below 0.05 °C, enabling the detection of extremely small local temperature variations [10].

The advantage of IRT over contact methods is that it provides instantaneous and sensor-based dynamic information about the temperature distribution without disturbing the natural thermal balance of the skin by attaching temperature sensors [11]. This enables the observation of temporal and spatial variations in surface temperature caused by external factors (e.g., wearing a protective face mask), as well as the analysis of the rate of heat accumulation and dissipation [12].

IRT is recognised as a reliable tool for the objective evaluation of thermophysiological comfort, including in studies of textile materials [13,14], sportswear textiles [15], chairs [16] and personal protective equipment [17,18]. Besides high sensitivity and non-contact nature, it enables the visualisation of local temperature zones on the body surface and the evaluation of heat exchange processes between the skin and the environment under real conditions [19,20].

Wearing protective face masks creates a localised microenvironment in which the exchange of heat and moisture between the skin beneath the mask and the surrounding air is restricted [21]. As a result, temperature and humidity increase under the mask, which is perceived as thermal and physiological discomfort [22,23]. Despite the numerous studies focusing on the final temperature values when using masks, the dynamic evolution of facial temperature during mask donning, prolonged wear, and removal remains insufficiently investigated. This temporal component is crucial for assessing the adaptive capacity of the skin and the degree of thermal load during mask wearing.

The application of IRT in this context offers a sensor-based experimental approach for the quantitative evaluation of thermal discomfort. The method allows the tracking of temporal characteristics of the process, such as how rapidly heating occurs when the mask is put on and how the thermal load evolves after its removal. IRT is able to capture spatial irregularities in heat accumulation, for instance in areas around the nose, lips, and eyebrows [24], where the mask fits most tightly and the thermal load is most pronounced [8,25]. IRT also makes it possible to analyse uncovered facial areas, such as the forehead and the eye corners, which indirectly respond to thermal stress [26] and reflect the dynamic reactions of the thermoregulatory system [27].

The present study investigates the applicability of IRT as a non-contact sensing tool for tracking dynamic changes in facial temperature during and after wearing different types of face masks. By means of a quantitative analysis of temporal temperature variations, the study focuses on characterising dynamic facial thermal response under controlled conditions, rather than relying solely on static temperature values. The results highlight the suitability of IRT for comparative, time-dependent evaluation of facial thermal behaviour associated with mask wearing and support its use in experimental sensor-based investigations.

## 2. Materials and Methods

### 2.1. Mask Samples and Physical Characterisation

**Figure 1:**
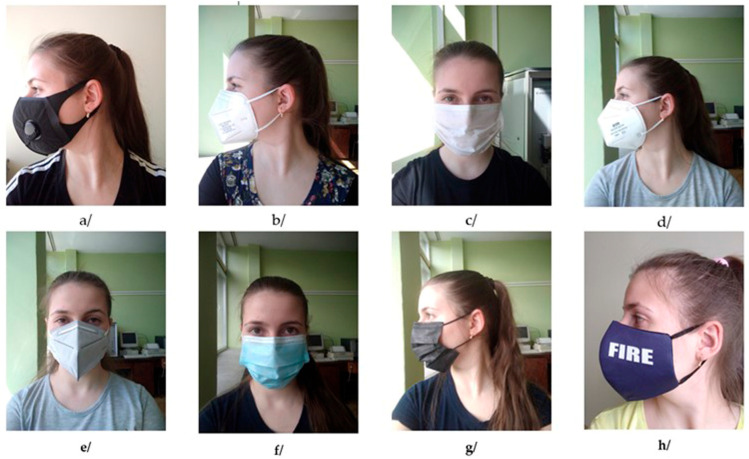
The investigated masks: (**a**) Sample A, respirator, (**b**) Sample B, respirator, (**c**) Sample C, surgical mask, (**d**) Sample D, respirator, (**e**) Sample E, respirator, (**f**) Sample F, surgical mask, (**g**) Sample G, surgical mask, (**h**) Sample H, textile mask.

The investigated masks were chosen to represent the most common categories used in public and clinical settings, enabling comparative, sensor-oriented evaluation of their structural and thermal performance. The selection, shown in Figure 1, encompassed four commercially available filtering respirators (labelled A, B, D, and E), three standard surgical masks (samples C, F, and G) and one reusable textile face mask (sample H), distinguished by its hybrid construction (knitted textile combined with a non-woven filter layer).

The physical parameters of the masks—thickness and bulk density—were preliminarily determined using precision laboratory instruments under controlled conditions.

Thickness δ is one of the main parameters that determine the functional and comfort characteristics of face masks. It directly affects the heat exchange between the facial skin and the surrounding environment: thicker masks usually have higher thermal resistance and retain more heat in the microclimate beneath the mask [28]. This expectedly leads to an increase in local skin temperature and creates a feeling of thermal discomfort. At the same time, thickness also influences air transfer—masks with greater thickness restrict air movement, which increases breathing resistance [29]. On the other hand, greater thickness is often associated with better filtration efficiency [30], as particles pass through a longer and more complex path within the material structure. Therefore, measuring thickness is essential for the sensor-based interpretation of thermal behaviour and the dynamics of heat accumulation and dissipation during wear.

The thickness of each sample was recorded using a mechanical thickness gauge (model DD-200-T, Hans Schmidt & Co GmbH, Waldkraiburg, Germany).

Bulk density $\rho_{b}$ is an indicator that reflects the amount of mass contained within the volume of the mask, including the air trapped between the fibres (or yarns, in the case of Mask H) within each layer, as well as the air gaps between the individual layers. It characterises the compactness of the structure and is directly related to the mask’s thermal insulation, air permeability, and filtration properties [31]. Masks with lower bulk density contain more air and generally provide better thermal insulation and higher breathability, whereas masks with higher bulk density are more compact and exhibit better filtration efficiency but may lead to greater heat accumulation and a sensation of discomfort [23].

The measurement of bulk density allows the assessment of the degree of compromise between these properties and helps to determine the balance between protection and comfort. It is calculated using the equation [32]:

(2) $$\rho_{b}=\frac{m}{A\cdot \delta }$$

where m is the mass of the mask (kg); A is the area of the mask (m^2^) and δ is mask’s thickness (m).

The mass of the masks was measured with a high-accuracy electronic balance (model KERN ABJ-NM/ABS/N, Kern & Sohn, Balingen, Germany). The number of layers was determined through destructive analysis of the masks.

Table 1 summarises the type, material, number of layers, and key physical properties of the examined masks.

### 2.2. Participants and Experimental Environment

Three healthy volunteers (PhD students, aged 27–46) participated in the study. The measurements were conducted in accordance with the Declaration of Helsinki. Ethical review and approval were waived, as the procedures involved non-invasive infrared surface imaging without medical intervention. Prior to the thermal imaging, each participant signed an informed consent form and confirmed the absence of respiratory symptoms or active infections.

The measurements were carried out within one day for each participant to minimise physiological variability. The experiments were conducted in an office room at the Technical University of Sofia, at an air temperature of approximately 23 °C, maintained by an air conditioner without direct airflow toward the participant. Before each series of measurements, the room was ventilated for 30 min, after which the windows were closed to ensure a stable microenvironment.

According to local meteorological records, the average outdoor air temperature on these days was approximately 2.0 °C, 3.4 °C, and 3.5 °C, respectively, with relative humidity ranging between 70% and 74%. Although the measurements were performed indoors under controlled conditions, these data are reported to characterise the external climatic background during the experimental period.

Participants did not wear makeup or skincare products on the day of the experiment. Each subject spent at least 10 min in the room prior to the first measurement to allow thermal adaptation. During mask wearing, the participant remained seated and silent. After changing each mask, a 15-min break was provided to allow the facial skin to restore its thermal equilibrium. During the break, the participant remained in a sitting position.

To reduce possible effects of adaptation or fatigue of the participants and to ensure comparability of the study, the sequence of mask testing was kept identical for all participants, as follows: surgical masks (samples C, F, and G), respirators (D, E, A, and B), and the textile mask H.

### 2.3. Thermographic Measurement Protocol

Facial skin temperature was captured with an infrared thermal camera (FLIR E6, FLIR Systems Inc., Wilsonville, OR, USA). The camera features a thermal sensitivity below 0.06 °C and measurable temperature range between −20 °C and 250 °C, The infrared image resolution is 160 × 120 pixels (320 × 240 display resolution). The instrument allows emissivity adjustment within 0.1–1 and offers three measurement modes. All thermal images were taken from a distance of 0.5 m from the participant’s face.

**Figure 2:**
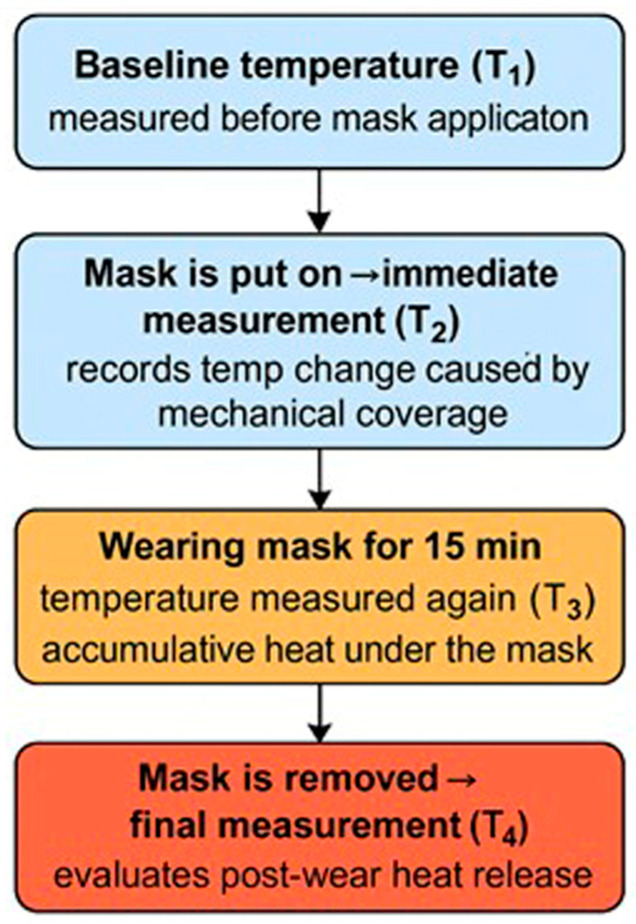
Flowchart of the thermographic measurement protocol.

The experimental sequence of thermal imaging during the mask tests is summarised in Figure 2. Four thermograms were captured for each mask: before application (T_1_), immediately after putting on the mask (T_2_), after 15 min of continuous wear (T_3_), and immediately after mask removal (T_4_).

This protocol enabled the evaluation of the dynamic thermal response of the facial skin throughout the phases of mask application, use, and recovery. The baseline temperature T_1_ served as a reference for the initial skin temperature prior to mask use. Measurement T_2_ recorded the immediate effect of mechanical coverage on skin temperature. Temperature T_3_ reflected the accumulated heat beneath the mask, as well as heat absorbed by the mask itself during wear. The final measurement, T_4_, indicated the release of retained heat after mask removal.

To quantify the thermal response of the facial skin during mask usage, four temperature-derived indices were defined and calculated based on the four measurement stages (T_1_–T_4_). These indices provide a structured view of time-dependent heat dynamics associated with the application, wearing, and removal of the face masks. A summary of the formulas and their interpretations is presented in Table 2.

All thermal images were analysed using FLIR Tools software (v. 5.0). Thermal data were extracted from ten predefined facial regions, as shown in Table 3. These regions included the entire face, nose area, cheeks, periorbital zones (inner eye corners), orbicularis oris muscle, depressor supercilii muscles (left and right), and the area covering the mask surface (measured only while wearing the mask). Each zone was marked manually with reference to consistent anatomical landmarks to ensure comparability across participants and masks.

The temperature at the inner canthus of the eye is recognised as one of the most stable facial points for assessing thermal variations. This is because the area is anatomically close to deep blood vessels and exhibits low sensitivity to fluctuations in the external environment. According to [33], it differs on average by 1.8 °C from the oesophageal temperature, yet remains a reliable indicator of core temperature dynamics. Another study [26] confirms that the inner canthus most accurately reflects changes in body temperature when the local thermal balance is altered. Therefore, in the present study, the left and right inner canthus were selected as reference facial regions not directly covered by the mask.

This methodological framework ensured reproducible, ethically compliant, and sensor-based dynamic evaluation of facial thermal responses to diverse mask structures.

## 3. Results

The results are interpreted within the framework of a dynamic thermographic protocol comprising four measurement stages (T_1_–T_4_), designed to capture facial thermal behaviour during mask application, prolonged wear, and post-removal recovery.

**Figure 3:**
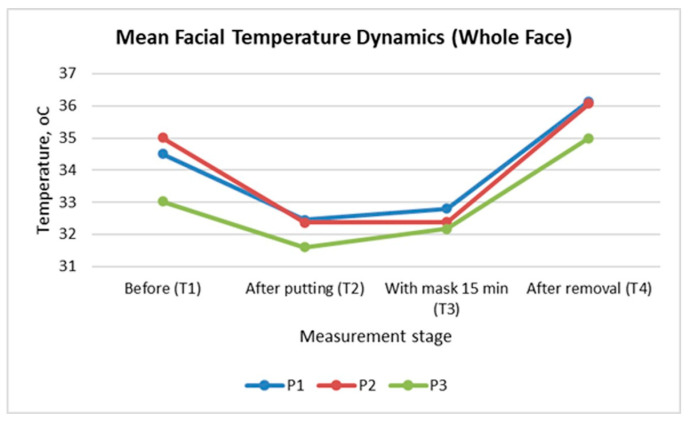
Mean facial temperature dynamics for the whole face during mask wearing and removal.

Figure 3 illustrates the dynamics of the mean facial temperature for the entire face across these four stages in the three participants. A similar pattern is observed for all participants: an initial decrease in temperature immediately after mask application (T_2_), followed by a slight increase after 15 min of wearing (T_3_), and a pronounced recovery (even exceeding the baseline values) after mask removal (T_4_).

This sequence reflects the typical dynamic thermal response of the skin when the face is covered. The sharp drop at T_2_ results from the mechanical isolation of the skin surface: the infrared camera registers the cooler surface of the applied mask. The increase at T_3_ indicates a gradual rise in heat beneath the mask and warming of the mask’s surface. The increment at T_4_ demonstrates the accumulated heat on the face resulting from the mask use and the subsequent recovery phase after removal.

Although the overall trend is similar, Participant 3 (P3) shows lower absolute temperature values at all stages. This outcome is likely due to individual differences in baseline skin temperature (T_1_), local blood perfusion, or mask fit. Participant 1 (P1) and Participant 2 (P2) exhibit almost identical thermal dynamics, with a distinctly higher value at T_4_ (approximately 1.5 °C above T_1_), indicating a stronger thermal response as a result of mask wearing.

**Figure 4:**
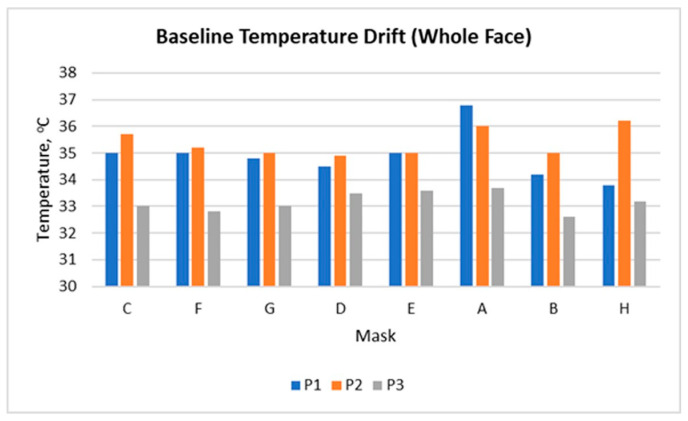
Baseline facial temperature drift during sequential mask testing.

Figure 4 shows the changes in the baseline temperature (T_1_) for the entire face in the three participants, measured before applying each of the eight masks. The data illustrate a drift in the baseline temperature throughout the test sequence, reflecting the dynamics of the personal thermoregulation during the experimental day.

Participants 1 and 2 display a similar pattern, with a gradual decrease in baseline temperature in the middle of the series followed by fluctuations. The highest values are recorded for the masks tested later in the sequence, indicating a cumulative thermal load and incomplete recovery of skin temperature between trials. Participant 3 shows a more stable line with minimal deviations, suggesting more efficient thermoregulation or lower sensitivity to heat accumulation. Another explanation could be again the face shape that supposes a looser fit of the investigated masks.

These results confirm that even after 15-min breaks, facial thermal equilibrium is not fully restored between successive measurements. This suggests that during prolonged or cyclic mask wearing (repeated donning and removal throughout the day), residual heat may accumulate and fail to dissipate completely, thus leading to a gradual rise in local skin temperature and potential thermal load.

**Figure 5:**
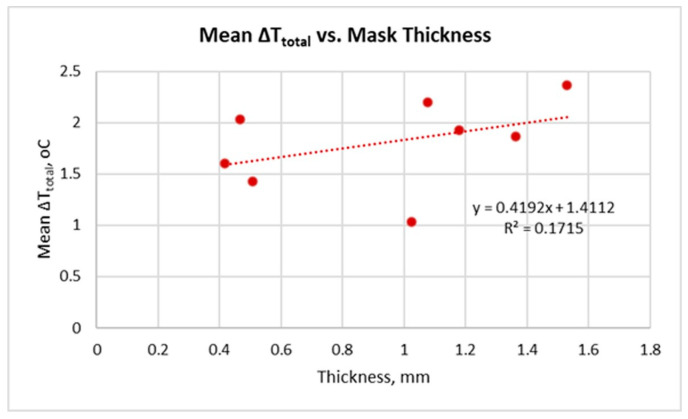
Mean total facial temperature change (ΔT_total_ = T_4_ − T_1_) in relation to mask thickness.

Figure 5 shows the relationship between the mean ΔT_total_ and mask thickness. The parameter ΔT_total_ is defined as the difference between the facial temperature after mask removal (T_4_) and the temperature immediately before mask application (T_1_)—see Table 2. mean ΔT_total_ represents the arithmetic average of this parameter for the three participants.

The data show positive mean ΔT_total_ values for all masks: the facial temperature after mask removal is higher than immediately before application. This confirms that during mask wearing, heat accumulates in the skin beneath the mask, leading to a short-term facial warming effect.

The linear regression demonstrates a moderately positive relationship between mask thickness and Mean ΔT_total_ (R^2^ = 0.1715). Therefore, thicker masks tend to retain more heat and cause a more pronounced thermal effect. This is consistent with the expectation that greater thickness increases thermal resistance and reduces the potential for heat dissipation through the material.

**Figure 6:**
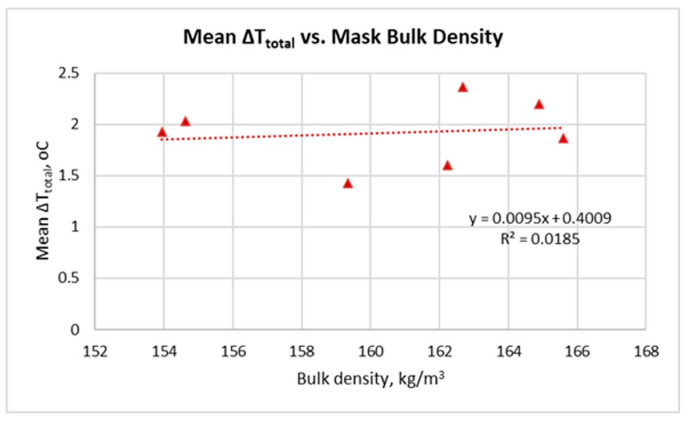
Mean total facial temperature change (ΔT_total_ = T_4_ − T_1_) in relation to mask bulk density (excluding mask H).

The relationship between the mean ΔT_total_ and the bulk density of the masks is shown in Figure 6. Mask H was excluded from the calculations because its structure and bulk density value (306.6 kg/m^3^) differ significantly from the other samples, forming a separate cluster that obscures the trend among the remaining masks.

The data show that even after excluding mask H, the relationship between bulk density and mean ΔT_total_ remains weak (R^2^ = 0.0185). The slight positive tendency suggests that denser masks retain slightly more heat, but the effect is minimal and should be interpreted cautiously given the limited sample size and narrow density range. This indicates that within the examined range of 154–166 kg/m^3^, the bulk density of the mask does not have a substantial impact on heat accumulation during wear.

**Figure 7:**
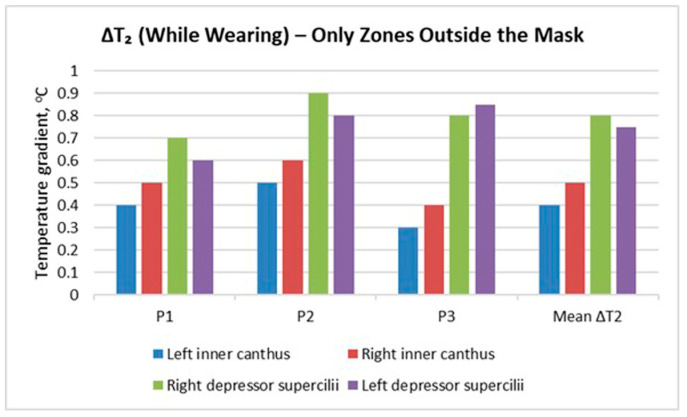
Temperature gradient during mask wearing (ΔT_2_) in facial zones outside the mask area.

Figure 7 presents the temperature gradient values ΔT_2_, calculated during mask wearing (T_3_ − T_2_), but only for facial zones not covered by the mask. Four sensitive regions were analysed: the inner corners of the eyes (left and right canthus) and the areas above the depressor supercilii muscles (left and right).

The results show that all participants experienced an increase in temperature, even though these areas were not directly covered by the mask. This increment was most noticeable in Participant 2 (P2), particularly in the right depressor supercilii region (around 0.9 °C). This may indicate a stronger thermal response, but it is also possible that the mask was not perfectly symmetrical, resulting in closer contact between the mask edge and the skin on that side.

The mean values (Mean ΔT_2_) confirm a general trend of moderate temperature rise across all observed areas, with the highest values around the eyebrow region (depressor supercilii). This suggests that the heat accumulated beneath the mask is transferred to adjacent facial zones through conduction and changes in air circulation around the face.

These results highlight that the thermal effect of mask wearing is not limited to the covered area but also affects peripheral facial regions, which should be considered when evaluating overall thermal response.

**Figure 8:**
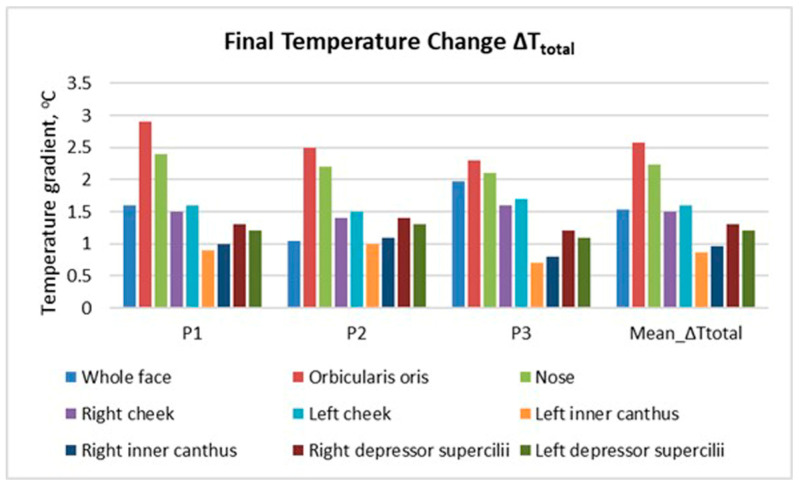
Final facial temperature change (ΔT_total_ = T_4_ − T_1_) across facial zones after mask removal.

Figure 8 presents the final facial temperature change (ΔT_total_ = T_4_ − T_1_) across the examined facial zones for the three participants, as well as the mean value for all zones.

The results show a clear increase in temperature after mask removal. The highest values are recorded in the orbicularis oris (around the mouth) and nasal regions. These areas are in direct contact with the mask, beneath which the largest amount of heat accumulates during wear. Elevated values are also observed in the depressor supercilii region, which is not covered by the mask but is indirectly affected by heat transfer and altered air circulation around the upper part of the face.

The mean values (mean ΔT_total_) confirm the trend of temperature increase across all analysed regions, with the highest rise in the central facial zones (nose, mouth, cheeks). The peripheral areas (the inner eye cantus and cheeks) show a more moderate thermal response. This distribution reflects the uneven heat accumulation across the facial surface.

The obtained results demonstrate that heat accumulated beneath the mask dissipates towards the peripheral facial areas not covered by the mask. Nevertheless, the overall facial heat load during mask wearing is evident, indicating measurable thermal loading.

**Figure 9:**
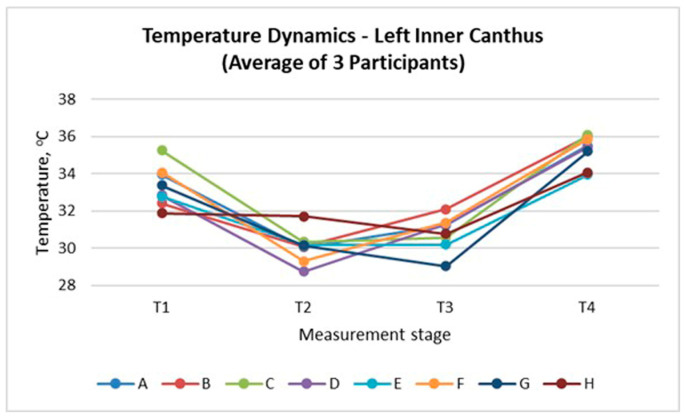
Temperature changes at the left inner canthus illustrating the thermal response to mask wear (average of three participants).

**Figure 10:**
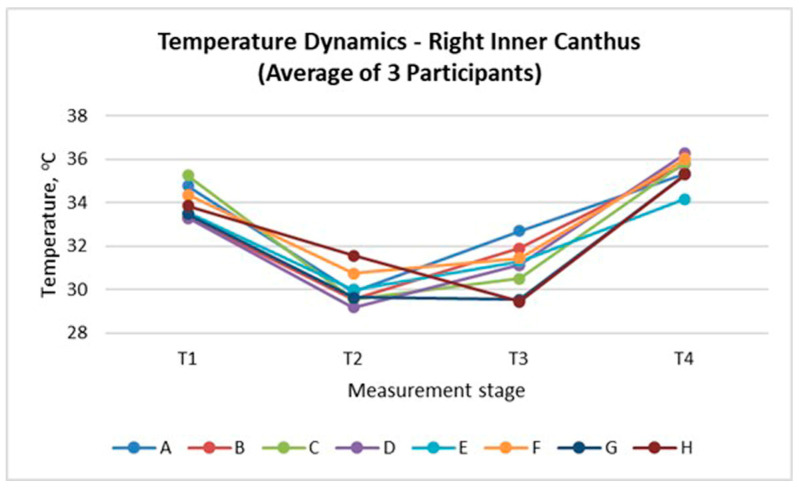
Temperature changes at the right inner canthus illustrating the thermal response to mask wear (average of three participants).

The inner canthus of the eye serves as an appropriate reference point for evaluating thermal responses, as it is not in direct contact with the mask but remains influenced by the altered facial microenvironment. Figure 9 and Figure 10 present the temperature dynamics at the left and right inner canthus, respectively. A consistent pattern was observed across all mask types, characterised by an initial temperature decrease immediately after mask application (T_2_), partial recovery during wear (T_3_), and a distinct rise after mask removal (T_4_). The largest drop at T_2_ occurred for Masks D and E, whereas the highest final values at T_4_ were registered for Masks C and E, indicating stronger heat accumulation. In contrast, the textile Mask H showed the smallest overall variation, suggesting a more balanced thermal behaviour. The similarity between both sides confirms the bilateral symmetry of the thermal response and highlights the sensitivity of the inner canthus as a reliable indicator of local thermal adaptation.

**Figure 11:**
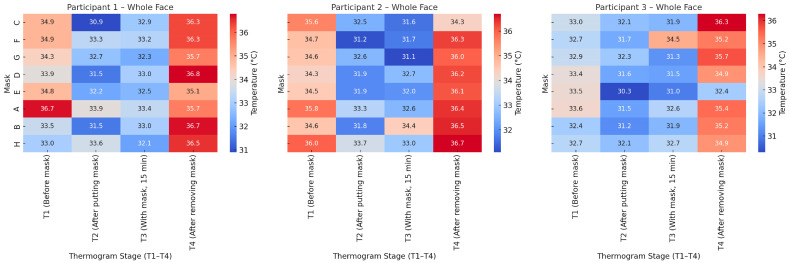
Heat maps of whole-face temperature for the three participants at four thermogram stages (T_1_–T_4_).

The heat maps in Figure 11 illustrate the temperature distribution for the whole face across the four thermogram stages (T_1_ ÷ T_4_) for the three participants. In all cases, a distinct temperature decrease is observed immediately after mask placement (T_2_), followed by a moderate increase during mask wear (T_3_) and a marked rise after mask removal (T_4_).

Participant 1 exhibits the largest temperature drop at T_2_, particularly for Masks C and D, and the most visible recovery at T_4_. Participant 2 shows smaller variations between stages, but overall higher final temperatures after mask removal, especially for Masks B and H. Participant 3 demonstrates the most balanced temperature profile, with smaller fluctuations across all stages.

Despite individual differences, the overall trend confirms a consistent thermal dynamic response: an initial cooling effect induced by mask application, followed by progressive warming and full temperature restoration after removal.

**Figure 12:**
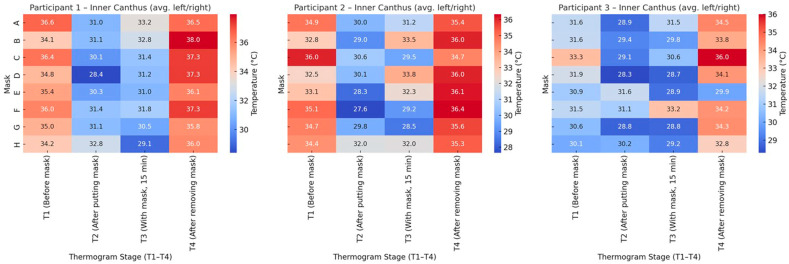
Heat maps of inner canthus temperature (average of left and right) for the three participants at four thermogram stages (T_1_–T_4_).

Figure 12 presents the temperature distribution at the inner canthus (average of left and right eye) for the three participants. The thermal pattern closely follows that observed for the whole face: a marked temperature drop immediately after mask placement (T_2_), partial recovery during wear (T_3_), and a distinct increase following mask removal (T_4_).

For Participant 1, the temperature decline at T_2_ was the most pronounced for Masks D and E, while Masks C and F reached the highest post-removal values (T_4_), indicating stronger local warming. Participant 2 displayed slightly higher overall temperatures and a sharper rise at T_4_, particularly for Masks E and H, suggesting more efficient heat retention. Participant 3 exhibited lower baseline values and smaller differences between stages, reflecting a more stable thermoregulation.

Across all participants, the inner canthus zone showed greater sensitivity to the microclimatic changes due to mask wearing. Its temperature variations mirror the overall facial response, confirming this region as a reliable local indicator of thermal adaptation and recovery.

**Figure 13:**
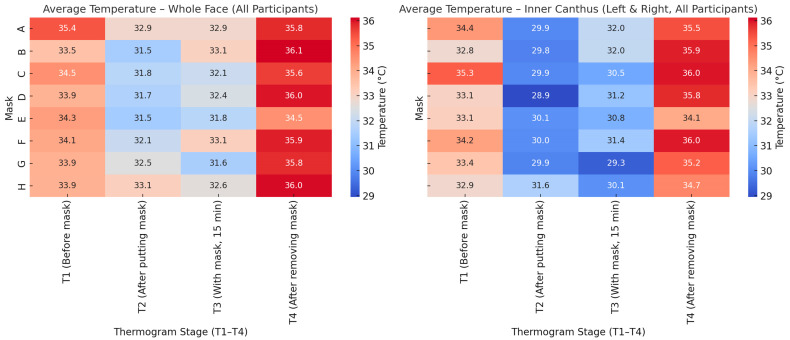
Comparison of average temperature dynamics for the whole face and inner canthus (left + right) across all participants at four thermogram stages (T_1_–T_4_).

The comparative heat maps in Figure 13 illustrate the average facial and inner canthus eyes temperature responses for all participants. Both the whole-face and inner canthus regions follow a similar trend, characterised by an immediate temperature decrease after mask application (T_2_), gradual recovery during wear (T_3_), and a marked rise after mask removal (T_4_).

The inner canthus shows a more pronounced temperature contrast between stages, indicating its higher sensitivity to facial microenvironmental changes. While the whole-face pattern reflects the overall heat accumulation beneath the mask, the inner canthus responds faster and more sharply, capturing transient thermal shifts. This difference highlights the importance of combining global and local thermal indicators when assessing the impact of mask use on facial thermoregulation.

## 4. Discussion

### 4.1. Dynamic Facial Thermal Response During Mask Application, Wear and Removal

The results obtained (Figure 3, Figure 4, Figure 5, Figure 6, Figure 7, Figure 8, Figure 9, Figure 10, Figure 11, Figure 12 and Figure 13) reveal a consistent dynamic pattern of facial temperature response to mask use across all participants, characterised by an initial cooling phase followed by progressive warming during wear and recovery after removal. This reproducible temporal pattern was observed across different mask types and participants, indicating a stable dynamic response rather than isolated individual effects.

It should be noted that the baseline temperature (T_1_) did not fully return to its initial level between successive measurements (Figure 4). This drift reflects the cumulative thermal load associated with repeated mask wearing rather than a methodological artefact. For this reason, all ΔT values were interpreted relative to each mask’s own baseline instead of being compared as absolute values across masks. This approach was intentionally adopted to preserve the physiological relevance of the observed temperature drift, as no correction was applied, since the drift itself represents a meaningful physiological pattern relevant to real cyclic mask use. Nevertheless, the limited sample size should be considered when interpreting these trends.

The initial decrease in T_2_ is mainly due to the conductive heat exchange between the skin and the mask itself, the temperature of which corresponds to the ambient temperature. When put on, the mask absorbs part of the heat from the face until thermal equilibrium with the skin is reached. This leads to a short-term cooling of the surface, recorded by the thermal camera. After the first minutes of wearing, heat accumulation begins in the microenvironment between the face and the mask, which leads to a gradual increase in the local temperature (T_3_). After removing the mask (T_4_), a rapid recovery and even exceeding the initial temperature is observed, which may be associated with the restoration of airflow and perfusion-related thermoregulatory adjustments.

The increase in the temperature of the face (T_4_) after removing the mask is observed not only in the areas that were covered, but also in the uncovered areas. This phenomenon can be explained by the interaction of several sequential physical and physiological mechanisms that are activated simultaneously. It is known that while wearing the mask, a local microenvironment climate is formed between the face and the mask—a limited air space with high humidity and elevated temperature. The skin constantly releases heat that cannot be effectively dissipated by convection, and the accumulated moisture from the exhaled air increases the heat capacity of the air layer. As a result, a partial thermal equilibrium is reached between the face, the mask and the air beneath it, which can be described as a greenhouse effect. Under these conditions, the temperature of the skin surface gradually increases, limiting natural heat dissipation.

When the mask is removed, the thermal system is abruptly disrupted. The warm, humid air trapped beneath it is replaced by cooler, drier air from the environment. This causes intense convection and evaporation of moisture from the skin, which initially has a cooling effect. At the same time, however, the body responds to the preceding thermal load by changes in superficial skin perfusion. Such perfusion-related responses may contribute to the observed increase in the measured skin temperature. Additionally, the thermal camera no longer registers the cooler surface of the mask, but the actual skin temperature, which also contributes to the reported increase in T_4_.

The increase in temperature in uncovered areas, such as the inner canthus of the eye and the area above the eyebrows, is due to two interrelated processes. On the one hand, the face is a common thermoregulatory system with an interconnected blood supply, in which local warming in one area affects neighbouring areas. On the other hand, the restoration of airflow after removing the mask leads to rapid temperature equalisation through convective heat exchange between different areas of the face.

The combined effect of the release of stored heat, perfusion-related thermoregulatory adjustments, and the change in convection explains the observed increase in temperature (T_4_) in the entire facial region after removing the mask. This behaviour represents the final stage of the thermoregulatory system’s response aimed at restoring thermal equilibrium after the period of isolation.

Monitoring the baseline temperature T_1_ before each mask (Figure 4) shows that thermal equilibrium is not fully restored even after a 15-min rest between measurements. This suggests that with prolonged or cyclical wearing of masks (alternately putting on and taking off), a cumulative thermal effect may occur, leading to a gradual increase in skin temperature and a feeling of thermal discomfort. This observation highlights the importance of dynamic rather than purely static thermal assessment.

A limitation of the protocol is the use of a fixed mask sequence. Although chosen to maintain controlled structural progression, this approach introduces an order effect. The observed upward drift in T_1_ reflects this accumulated load. Future studies should adopt randomised or balanced experimental designs and include a larger and more diverse participant cohort to separate sequence-related and structure-related effects more clearly.

### 4.2. Influence of the Structural Parameters of Masks on Thermal Dynamics

The relationship between the morphological characteristics of masks and the thermal effect on the face is essential for understanding the observed temperature changes.

The results suggest that material thickness shows a general positive tendency toward higher heat accumulation under the mask, which coincides with the findings in [28]. As shown in Figure 5, the positive relationship between the thickness and the average value of ΔT_total_ (T_4_ − T_1_) confirms that thicker and multi-layered masks limit heat transfer and retain more heat in the skin [34]. The temperature increase after removing the mask is more pronounced, which indicates a higher degree of heat accumulation during wearing. This relationship is expected, since with rise in layer thickness, the thermal resistance increases and the possibility of convective and diffusion heat exchange between the internal and external environments decreases.

The bulk density, on the other hand, has a weaker and practically limited effect. Figure 6 shows that in the considered range of bulk density (154 ÷ 166 kg/m^3^) its influence on ΔT_total_ is minimal (R^2^ = 0.0185). The weak positive trend suggests that denser materials can retain slightly more heat, but the effect is weak and does not significantly change the overall thermal response.

An interesting exceptional case is the textile mask H, which was excluded from the regression analysis due to its significantly higher bulk density (306.6 kg/m^3^) and distinct structure. Nevertheless, the thermographic measurements indicate a more balanced thermal behaviour and a smaller temperature contrast between the measurement stages. This behaviour can be attributed to its textile morphology, with knitted outer layers that enhance air permeability and convective heat exchange, partially compensating for the higher material density.

These results confirm that the thermal behaviour of masks depends not only on their numerical parameters (thickness, density), but also on the internal structure of the material, the degree of porosity and the arrangement of the fibres. Masks with a more open and air-permeable structure allow better air circulation and limit overheating, while more compact and multi-layered structures lead to heat accumulation and increased skin temperature after removal.

It should be noted that, due to the exploratory nature of the study and the limited number of participants, the reported relationships are interpreted as tendencies rather than statistically significant correlations.

### 4.3. Heat Transfer to Peripheral (Uncovered) Areas

The results of thermographic measurements show that wearing a mask causes changes in the temperature profile not only of the covered areas, but also of the peripheral facial areas that are uncovered by the mask. Figure 7, Figure 8, Figure 9 and Figure 10 clearly demonstrate an increase in temperature in the areas above the eyebrows (depressor supercilii) and in the inner canthus of the eyes, despite the fact that these areas are not beneath the mask.

The reason for this phenomenon is the combined action of two mechanisms—thermal conduction through the tissues and changes in the local convection of the air around the face. The heat accumulated under the mask is transferred to the skin through the surface layers and the capillary network. The increased blood flow, activated by local overheating [35], leads to dilation of the vessels in the adjacent uncovered areas as well. This creates a secondary warming zone that includes the eye and forehead regions.

The second factor is related to the change in air movement around the face. The mask redirects exhaled air upwards (towards the eyes and temples) [36], especially in mask models with a stiffer construction or a loose fit along the nose line. The warm and humid airflow increases the skin temperature in these areas and contributes to local warming even outside the contact area [8]. This phenomenon is most pronounced in participant 2, whose thermographic data show an asymmetric increase in temperature in the right forehead area, probably due to a slight lateral air leak from the mask.

The combination of these two mechanisms—conduction and redirected convection—explains why the inner canthus and the area above the eyebrows respond with a moderate rise in temperature during wear. This confirms that facial thermoregulation acts as an interconnected system in which local changes under the mask trigger an adaptive response in the uncovered areas as well.

The results obtained emphasise the need to include peripheral areas in the assessment of thermophysiological comfort, as they reflect both direct heat accumulation and secondary effects from airflow and vascular response. In this context, the inner canthus area represents a reliable indicator of the overall thermal state of the face, as it registers mechanically transferred and physiologically induced heat.

### 4.4. The Role of the Inner Canthus as a Local, Sensitive Indicator of Thermoregulation

The inner canthus area of the eyes occupies a special place in the analysis of the thermophysiological responses of the face. The reason is that it combines two key characteristics: it is not directly covered by the mask, but is located in close proximity to its upper border. This position makes it extremely sensitive to changes in the local microclimate and to the physiological adaptations of the body to the heat load.

The results of Figure 9, Figure 10, Figure 11, Figure 12 and Figure 13 show that the temperature dynamics in the inner canthus area follows the same pattern observed for the entire face—a decrease at T_2_, partial recovery at T_3_ and a distinct increase at T_4_. However, the amplitude of the fluctuations is larger. This means that the area reacts faster and more intensely to both the initial cooling and the subsequent warming. In all participants, symmetry of the response between the left and right eyes was recorded, which indicates a stable physiological basis of the process, and not a random variation.

Physiologically, this behaviour can be explained by the rich blood supply and thin skin in the periorbital region [37]. The inner canthus is connected to a dense capillary network that reflects changes in skin perfusion in almost real time [38]. Thus, when heat accumulates under the mask and vasomotor mechanisms for heat dissipation are activated, this area responds with an increase in temperature, even without direct contact with the heat source. At the same time, when the mask is put on, the cold material lowers the temperature of the face by conduction, and the cooling is quickly reflected in this area due to the thermal connectivity of the vascular system. These observations are consistent with the findings of [33], according to which the temperature of the inner canthus reflects the fluctuations of the oesophageal temperature with minimal delay. A similar conclusion is presented in [35], where the inner canthus is defined as the most stable and reliable facial point for monitoring core body temperature. The present results confirm these data and add that when wearing a mask, the inner canthus functions as a reactive thermal indicator of changes in local thermoregulation.

The importance of this area is also enhanced by its independence from mechanical contact with the mask. It provides an opportunity to track secondary thermal effects: how the heat accumulated under the mask affects the rest of the facial areas. Its position near the eyes also makes it an indicator of subjective comfort, as temperature fluctuations in this area are associated with sensations of dryness, burning and eye strain, often reported during prolonged mask wearing [39].

In this context, the inner canthus can be considered as a reliable indicator for assessing thermoregulation in experiments related to facial microclimate. Its inclusion as a recommended area for thermographic analysis would allow for more accurate and localised tracking of the dynamics of heat stress and recovery after wearing a mask.

### 4.5. Assessment of Thermophysiological Comfort by Mask Type

The analysis of heat maps (Figure 11, Figure 12 and Figure 13) shows clear differences between the individual masks in terms of temperature dynamics and the balance between cooling and heat retention. Masks C, E and F, characterised by a greater thickness and a denser multilayer structure, show the highest values of temperature after removal (T_4_), which indicates the accumulation of heat beneath them during wear. These models create a pronounced microenvironment with limited air exchange and higher humidity, which may be associated with lower thermophysiological comfort during prolonged use.

Masks A and D, also of the respiratory type, show a moderate thermal effect—the temperature drop upon putting them on is more pronounced, but the subsequent recovery is smoother. This suggests a better balance between insulation and air permeability, especially during short periods of wear.

The most balanced thermal response was demonstrated by the textile mask H, which in all participants showed the smallest temperature contrast between the individual stages (T_1_ ÷ T_4_) and stable values in the inner canthus area. Therefore, the mask allows for effective heat exchange with the environment and does not cause significant heat accumulation, which makes it the most favourable in terms of thermophysiological comfort.

Although subjective thermal comfort was not directly assessed in this study, the observed temperature patterns in both covered and uncovered facial regions provide an objective physiological basis that can be integrated with subjective comfort evaluation in future work.

### 4.6. Limitations

This study has some limitations that should be considered when interpreting the results. First, the number of participants was limited and the sample lacked demographic diversity. The study was therefore designed as an exploratory investigation, and the results are interpreted as descriptive trends rather than population-level statistical inference.

Second, a fixed mask testing sequence was used, which may introduce order effects reflected in the observed baseline temperature drift and interpreted as cumulative thermal load. Future studies should employ randomised or balanced designs to better separate sequence-related and structural effects.

Third, subjective thermal comfort was not assessed, as the small sample size would not allow statistically meaningful or generalisable evaluation. The integration of thermography with questionnaires or comfort scales is recommended for future studies involving larger cohorts.

Finally, the experimental protocol focused on short-term mask wearing under controlled indoor conditions. Prolonged wearing scenarios, outdoor environments, and real-world usage conditions were beyond the scope of the present study and are identified as important directions for future research.

## 5. Conclusions

The results of the present study show that the temperature changes on the face during protective mask use are determined by a complex interaction between the physical properties of the mask and the measured thermal behaviour of the facial skin. The initial cooling is caused by conductive heat exchange between the skin and the mask material, while the subsequent temperature increase results from heat accumulation in the local microenvironment beneath the mask and perfusion-related thermoregulatory adjustments of the skin.

Within the examined range of mask structures, no strong or unambiguous dependence of the thermal response on bulk density was observed, while material thickness showed only a limited tendency toward higher thermal insulation.

It was found that the thermal effect is not limited to the covered areas, but extends to the peripheral facial zones through conduction and redirected airflow. Among these, the inner canthus of the eye stands out as a sensitive and reliable local indicator of facial thermal behaviour. The combined analysis of the entire facial area and the inner canthus reveals the complete dynamics of thermal processes, from the initial cooling to the recovery phase after mask removal.

These findings illustrate the dynamic sequence of facial thermal response associated with mask wearing and demonstrate the applicability of infrared thermographic imaging as a non-contact sensing tool for dynamic assessment of facial temperature changes. Based on the observed temperature patterns, the textile mask showed a more balanced thermal response under the applied experimental conditions, whereas thicker and more compact mask structures were associated with greater heat accumulation.

## Figures and Tables

**Table 1:** Type, material, number of layers and corresponding standard of the investigated masks.

No.	Mask	Type	Layers	Mask Thickness, mm	Mask Bulk Density, kg/m^3^
1	Mask A	Respiratory	1	1.180	153.941
2	Mask B	Respiratory	5	1.363	165.604
3	Mask C	Surgical	3	0.465	154.626
4	Mask D	Respiratory	5	1.078	164.878
5	Mask E	Respiratory	6	1.530	162.679
6	Mask F	Surgical	3	0.417	162.236
7	Mask G	Surgical	3	0.507	159.351
8	Mask H	Textile	3	1.023	306.619

**Table 2:** Thermal indices calculated from infrared measurements.

Index	Formula	Meaning
ΔT_1_	T_3_ − T_1_	Apparent temperature change during wear (only for uncovered zones)
ΔT_2_	T_3_ − T_2_	Heat accumulation during mask application
ΔT_total_	T_4_ − T_1_	Total effect after mask removal: real change in facial skin temperature
TAI	ΔT_1_ + ΔT_2_	Thermal Accumulation Index: cumulative heat load during the entire mask-wearing period

**Table 3:** Defined facial zones used for thermal data extraction.

No.	Zones	Thermogram
1	Whole face	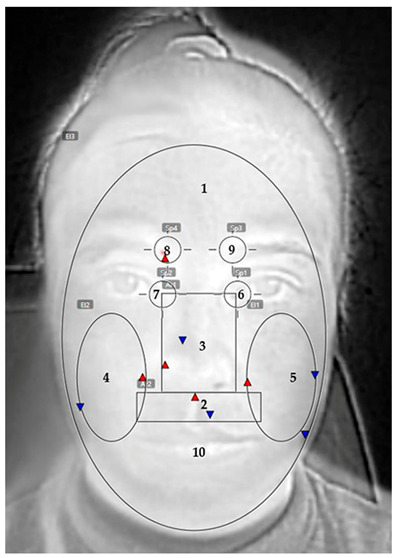
2	Orbicularis oris muscle (between the nose and the mouth)	
3	Nose zone	
4	Right cheek	
5	Left cheek	
6	Left inner cantus eye	
7	Right inner cantus eye	
8	Right depressor supercilii muscle	
9	Left depressor supercilii muscle	
10	Mask zone (only when mask is used)	

## Data Availability

The original contributions presented in this study are included in the article. Further inquiries can be directed to the corresponding author.
